# White Matter Hyperintensities on Admission CT Are Associated with Worse Outcomes but not Cerebral Edema or Hemorrhagic Transformation after Large Vessel Occlusion Stroke

**DOI:** 10.21203/rs.3.rs-9547399/v1

**Published:** 2026-05-19

**Authors:** Sophia Dietz, Amrit Avula, Atul Kumar, Marina Guasch-Jimenez, Pol Camps-Renom, Agnieszka M Slowik, Pawel Wrona, Lily Pwint Thinzar, David Vargas, Nils H Petersen, Rajat Dhar

**Affiliations:** Washington University School of Medicine in Saint Louis: Washington University in St Louis School of Medicine; Washington University School of Medicine in Saint Louis: Washington University in St Louis School of Medicine; Washington University School of Medicine in Saint Louis: Washington University in St Louis School of Medicine; Hospital de la Santa Creu i Sant Pau; Hospital de la Santa Creu i Sant Pau; Jagiellonian University in Kraków Medical College: Uniwersytet Jagiellonski w Krakowie Collegium Medicum; Jagiellonian University Medical College: Uniwersytet Jagiellonski w Krakowie Collegium Medicum; Yale School of Medicine: Yale University School of Medicine; Yale School of Medicine: Yale University School of Medicine; Yale School of Medicine: Yale University School of Medicine; Washington University School of Medicine in Saint Louis: Washington University in St Louis School of Medicine

## Abstract

**INTRODUCTION:**

White Matter Hyperintensities (WMH) are key radiographic biomarkers of cerebral small vessel disease (CSVD) and have been associated with worse outcomes after stroke. Since CSVD may involve blood-brain barrier disruption, we hypothesized that worse outcomes in those with WMH may be mediated through more severe cerebral edema and hemorrhagic transformation.

**METHODS:**

In this retrospective multicenter cohort study, we studied patients from four multinational stroke cohorts who presented within 12 hours of anterior circulation large vessel occlusion (LVO) stroke and received at least one follow-up CT between 12–48 hours. WMH were rated on baseline CTs using a Fazekas-derived system, with a subset also assessed with Fazekas-grading on MRI. Automated image analysis measured cerebrospinal fluid displacement (ΔCSF) and lesional-to-contralateral hemispheric CSF volumes (CSF-ratio) as quantitative edema biomarkers. We analyzed the association between WMH presence and edema severity, hemorrhagic transformation and poor functional outcome (mRS 3–6), adjusting for key covariates, overall and in the subgroup undergoing thrombectomy and those with successful reperfusion.

**RESULTS:**

Of 1,290 LVO patients, 782 were eligible for imaging analysis. Of these, 19% had WMH on CT. Patients with WMH were older, more often female and had a history of hypertension, and higher presenting systolic blood pressure and glucose levels. WMH presence was associated with worse functional outcome, adjusting for age and additional covariates [OR 1.84 (1.06–3.22), p = 0.04] but lower risk of any HT [OR 0.48 (0.25–0.88), p = 0.03]. However, WMH presence by CT or MRI was not associated with edema (ΔCSF, CSF-ratio, or midline shift) in the whole population or thrombectomy/reperfusion subgroups. Reperfusion was associated with less edema and improved recovery.

**CONCLUSIONS:**

Presence of CT-graded WMH was associated with worse outcome after LVO stroke, independent of age and other factors. However, this risk appears not to be mediated through greater risk of HT or cerebral edema formation.

## Introduction

White matter hyperintensities (WMH), are a common yet underappreciated radiographic indicator of cerebral small vessel disease with growing clinical relevance. Characterized by hyperintense signal on T2-weighted magnetic resonance imaging (MRI),^1^ WMH lesions represent areas of lost white matter structural integrity, arteriole stenosis, and increases in microglia and blood-brain-barrier (BBB) leakiness.^2,3^ These lesions are strongly associated with aging, with some studies suggesting that they are present, on average, in 11–21% of patients 64 years old and up to 94% of individuals aged 82 and older,^4^ and have been linked to neurodegenerative disorders, dementia, gait disturbances, and executive function decline.^2,5,6^

Growing evidence highlights their clinical relevance in the context of cerebrovascular disorders. More specifically, studies have found that increased WMH burden is associated with worse cognitive and functional outcomes after stroke,^7,8^ but the mechanistic basis for this association is poorly understood. One possible explanation of emerging interest is cerebral edema (CED), as pathological studies of WMH have revealed that WMH is associated with endothelial/venous damage, dysfunction of the glymphatic pathway, and ischemia-hypoperfusion,^9^ characteristics that are also implicated in the formation of cerebral edema and hemorrhagic transformation after ischemic stroke. These factors are especially relevant in the setting of large vessel occlusion (LVO) strokes, with increasing attention paid to these complications after endovascular thrombectomy (EVT) with successful or failed reperfusion.^10^ In fact, a reduction in edema has been proposed to mediate a majority of the association of successful vessel recanalization and improved outcomes.^11,12^

A recent study suggested that WMH burden on MRI was associated with higher risk of malignant cerebral edema (MCE) and poor functional outcomes post-EVT. However, this analysis used a dichotomized MCE definition based on midline shift, which may underestimate clinically meaningful edema progression.^13^ While MRI remains the gold standard for WMH detection, there is a pragmatic rationale for using computed tomography (CT) to evaluate WMH in the stroke setting given that decisions about early diagnosis and management in stroke are commonly based on CT.^14^

The present study aims to evaluate the relationship between CT-graded WMH and CED formation using quantitative metrics that measure global and hemispheric displacement of cerebrospinal fluid as sensitive imaging biomarkers of brain swelling that can be extracted automatically from routine CT scans. Our hypothesis is that CT-visible WMH is associated with increased post-stroke edema, hemorrhagic transformation, and worse functional outcomes.

## Methods

### Participant Selection

Acute ischemic stroke patients who were enrolled in cohort studies at four large centers between 2017 and 2024 were evaluated for inclusion in this retrospective analysis. We selected participants who presented within 12 hours of known stroke onset due to anterior circulation LVO (ICA or MCA occlusion), and who underwent at least one follow-up CT scan between 12–48 hours after stroke onset. Patients without follow-up scans were excluded. All de-identified data were shared with the coordinating institution for grouped analysis under appropriate data sharing and ethical approvals. The retrospective imaging analysis was approved by the institutional review board at the coordinating site with waiver of participant informed consent.

### Clinical Data

Baseline demographic data and timing of baseline and follow-up imaging data were collected. Additional clinical variables, including National Institutes of Health Stroke Scale (NIHSS) scores, presenting serum glucose and systolic blood pressure (SBP), history of smoking, hypertension (HTN), or diabetes mellitus (DM), and mRS at 90 days post-stroke were analyzed when available from each cohort.

### Neuroimaging Stroke and CED Data

Most participants underwent CT imaging on presentation and received a repeat CT around 24 hours after presentation or intervention. Follow-up scans performed closest to 24 hours after stroke onset were selected for analysis (range 12–48 hours). CT images were processed using an established automatic image analysis pipeline that uses machine learning to segment cerebrospinal fluid (CSF) regions and extract CSF volumes globally and in each hemisphere.^15^ Patients with poor CT scan quality or other segmentation errors were excluded from the sample. ΔCSF was calculated as the percent change in total CSF volume between baseline and follow-up scans and hemispheric CSF-ratio was calculated by taking the ratio of CSF volumes in each hemisphere (lesional vs contralateral).^16^ An exploratory distribution of ΔCSF was evaluated for any outliers and cases where CSF volume increased greater than 1.5 times the lower bound of the interquartile range (IQR) for ΔCSF were removed from the analysis. Net water uptake (NWU) was quantified on the follow-up CT scan by first segmenting regions of the infarct lesion and then calculating the relative density of this mask relative to the contralateral mirror region.^17^ Midline shift was measured at the septum pellucidum and hemorrhagic transformation (HT) was evaluated by one of two trained investigators using the European Cooperative Acute Stroke Study (ECASS) established grading system.^18^ HT was defined either as absent versus having petechial (HI-1/HI-2) or parenchymal hematoma (PH-1/PH-22), and severe cerebral edema was dichotomized as present or absent with respect to cases resulting in midline shift (CED grade 3).^19^

### WMH Assessment

WMHs were graded on baseline CT scans by one of two trained raters, a radiologist or a neurocritical care physician, who were blinded to clinical and subsequent imaging outcomes. Grading was performed using the Fazekas Scale, a method endorsed by the American Heart Association/American Stroke Association as a rapid and reliable method for grading WMH burden.^4^ Although originally developed for MRI, the Fazekas scale has been validated for use grading WMH on CT, with substantial interrater reliability.^14,20^ In the present study, we utilized a previously adapted Fazekas-derived grading system in which a total grade 0 to 3 was given in accordance with the extent and severity of WMH (0 = absent; 1 = capping of the ventricles or focal deep WMH; 2 = more extensive periventricular WMH or beginning of confluent deep WMH areas; 3 = confluent, irregular periventricular WMH extending to the cortex, or large confluent areas of deep WMH).^15^ Presence of WMH was defined as any Fazekas score above 0. An evaluation of inter-rater reliability was performed on 30 CT-scans graded independently and blindly by two raters. In addition, the same Fazekas scale was used to grade all available in-patient MRI scans from one cohort for WMH severity.

### Statistical Analysis

Univariate associations were conducted to examine to relationship among those with WMH presence and edema severity on FU CT. For linear regression, CSF ratio was transformed using the cube root of the original measurement subtracted from the maximal value (1.0) to satisfy the residual assumption and present normality to the distribution. To study the primary outcome, the effect of WMH was modeled after adjusting for age, baseline CSF volume, baseline NIHSS score, and vascular and metabolic factors selected based on established associations with WMH burden, cerebral edema, and stroke severity, including admission serum glucose, history of diabetes mellitus, hypertension, systolic blood pressure, tobacco use, and ASPECTS^7,13^. The influence of successful reperfusion was included as a covariate in those who underwent EVT. Variance inflation factor with a cutoff of 5 addressed multicollinearity of covariates. Nested comparisons were evaluated using the likelihood ratio test and F-statistic. The Benjamini-Hochberg method was used for multiple hypothesis testing and correct for the false discovery rate. For better interpretation of the outcome results, effect size of continuous predictor values were normalized per one standard deviation. A subgroup analysis was performed in those who underwent EVT and a sensitivity analysis after cases with parenchymal hemorrhage severity of HT were excluded due to the potential confounding on mass effect measurements for edema.

For secondary analysis, binary logistic regression models were used for specific patient outcomes using the same predictors of interest. The mRS variable was dichotomized where scores of 0–2 were considered a good functional outcome, and models were adjusted for the mentioned covariates. For all primary and secondary analyses, missing subject data was excluded from analysis, and imputation was not performed to avoid introducing bias in the pooled multicohort analyses. Similarly, patients lacking follow-up imaging data were excluded from analyses requiring follow-up CT.

### Artificial Intelligence

Artificial intelligence tools (ChatGPT 4.0 and OpenEvidence) were used solely for editorial improvements and preliminary literature identification. AI was not involved in data collection, study design, statistical analysis, or final bibliography generation.

## Results

### Patient Data and Cohort Characteristics

**Figure 1:**
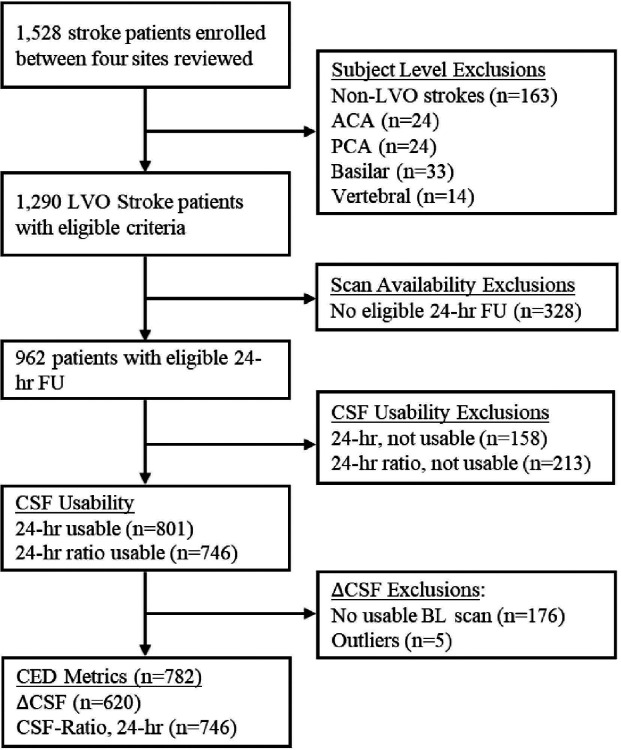
Flow of study participants eligible for analysis. In total, 782 patients had eligible data for inclusion in the ΔCSF and/or CSF Ratio analyses. The ΔCSF cohort required usable baseline and 24-hour CSF measurements. Of the 801 patients with usable 24-hour follow-up, 176 patients were excluded from the CSF cohort for baseline scan level issues, and 5 were excluded as CSF outliers (lower bound=−0.312), leaving 620 patients were included in the final ΔCSF cohort and 746 patients in the final CSF-ratio cohort. Abbreviations: ACA, Anterior Cerebral Artery; BL, Baseline; CSF, Cerebrospinal Fluid; ΔCSF, Change in Cerebrospinal Fluid; FU, Follow-up; LVO, Large Vessel Occlusion; PCA, Posterior Cerebral Artery

There were 1,290 stroke patients in the combined multi-center cohort. After exclusion of those without eligible follow-up scans, 962 patients underwent imaging analysis. A baseline CT was available in 665 (85%), performed at a median time of 1.9 hours (IQR: 1.2–3.5) from stroke onset, and all patients had a follow-up scan with a median time of 25.7 (IQR: 22.2–28.4) from stroke onset. Thrombectomy was performed in 605 (77%), of whom 489 (81%) had successful reperfusion (TICI 2B or greater). The final analysis included 782 who had one or both edema biomarkers successfully extracted (746 with measurable CSF ratio versus 620 with measurable ΔCSF, primarily missing due to lack of baseline or usable CT scan) (Fig. 1). Of the total cohort, 146 (19%) had WMH visible on CT. Of those with WMH visible, 81 (55%) had grade 1, 25 (17%) grade 2, and 17 (12%) grade 3. Testing of inter-rater reliability between two graders found a Cohen’s kappa of 0.60 for presence (p < 0.001) of WMH and weight Cohen’s kappa of 0.65 for severity of WMH (p < 0.001). In the cohort who underwent grading of WMH on MRI (available in 137 of 152 patients from one site), WMH were present in 110 (80%) on MRI, with half being grade 1, 37% grade 2 and 13% grade 3. The agreement of WMH from CT to MRI yielded a Kappa of 0.23 (p < 0.001) for severity and 0.09 (p = 0.01) for presence. Although this indicates weak agreement between modalities, subsequent patient characteristics and analysis primarily focused on CT-graded WMH as the primary population, with the MRI-graded cohort as a supplemental comparison.

Patients with WMH present on CT were significantly older, more likely to be female, had a higher median systolic blood pressure and higher serum glucose on arrival (Table 1). Those with WMH were more likely to have a history of hypertension but not higher chance of having diabetes. Additionally, patients with WMH were slightly less likely to receive EVT, but of those who underwent EVT, those with WMH were not less likely to achieve successful reperfusion than those without WMH. No significant differences were observed in baseline NIHSS or ASPECTS between the two groups (Table 1).

### Association between WMH and Post-Stroke CED and Hemorrhagic Transformation

Visible presence of WMH on CT was not associated with greater edema formation, as assessed by ΔCSF (p = 0.41) or CSF ratio (p = 0.20) (Table 1). Similarly, presence of any WMH was not significantly associated with other edema metrics including presence of midline shift (p = 0.28) or higher NWU at 24-hours (p = 0.57). In the MRI-graded cohort, WMH was similarly not associated with more severe edema. Additionally, severity of WMH was not associated with greater edema, either by CT or by MRI.

In multivariable linear regression, adjusting for potential confounders, ΔCSF was associated with NIHSS (p < 0.001), glucose (p < 0.001) and baseline CSF volume (p = 0.014), but not with age or WMH presence. Similarly, CSF-ratio was significantly associated with NIHSS (p < 0.001), glucose (p < 0.001), baseline CSF (p = 0.0398), and ASPECTS (p < 0.001), but not with age or WMH presence.

Repeating the analysis restricting to those undergoing EVT, we found that successful reperfusion was significantly associated with less edema formation on all three measures [ΔCSF: OR 0.91 (0.87–0.95), p < 0.001; CSF-ratio: OR 0.87 (0.82–0.92), p < 0.001; midline shift: OR 0.16 (0.06–0.43), p < 0.001], but WMH presence was not associated with edema in the EVT subgroup or the subset with successful reperfusion. WMH was also not a significant predictor for those with edema resulting in midline shift (CED-3). Similarly, WMH presence was not associated with edema in the sensitivity analysis when excluding patients with parenchymal hemorrhage. Interestingly, WMH was associated with a lower risk of any HT (29% vs 20%, p = 0.03). This association remained significant when adjusting for age, NIHSS, ASPECTS, and glucose [aOR 0.48 (0.25, 0.88), p = 0.03], but was attenuated in the EVT and reperfusion subgroups.

### The Impact of WMH on Functional Outcomes

**Figure 2.**
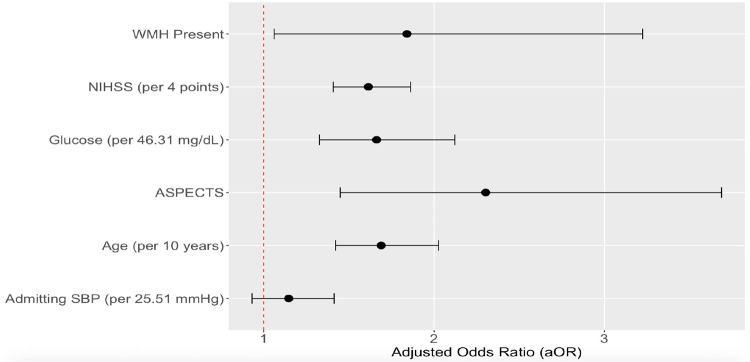
Associations of clinical and radiographic factors with stroke functional outcome. Forest plot showing the multivariate adjusted odds ratio (aOR) with 95% confidence intervals for association with 90-day modified Rankin Scale (mRS) of 3–6. Models were adjusted for age, WMH presence, baseline NIHSS, ASPECTS, glucose, and admission SBP. To aid comparability, effect sizes were normalized per standard deviation of each predictor variable; for glucose: 46.31 mg/dL; for admission SBP: 25.51 mmHg. Abbreviations: ASPECTS, Alberta Stroke Program Early CT Score; mRS, modified Rankin Scale; NIHSS, National Institutes of Health Stroke Scale; SBP, systolic blood pressure; WMH, white matter hyperintensity.

Presence of WMH on admission CT was significantly associated with worse functional outcome in univariate analysis [OR 2.56 (1.70, 3.90) p < 0.001]. Adding WMH to an outcome model with age, baseline NIHSS, ASPECTS, glucose, and admission SBP resulted in a lower AIC (587.42) than the baseline model (AIC 590.13). WMH remained a significant predictor for worsening functional outcome [aOR 1.84 (1.06, 3.22), p = 0.04] when adjusting for the aforementioned covariates (Fig. 2). In the cohort that analyzed WMH across both imaging modalities, WMH on MRI trended towards poor functional outcome (60% for WMH patients and 46% for non-WMH patients, p = 0.28), but was ultimately not associated with poor functional outcome [aOR 0.44 (0.10–1.65) for MRI]. However, CT-graded WMH in these same patients demonstrated a stronger trend to worse functional outcomes (aOR 1.81, 0.38–13.24). Reperfusion was associated with improved functional outcome in the EVT cohort [OR 0.14 (0.06, 0.30), p < 0.001], and WMH exhibited a non-significant trend towards worse functional outcome in this smaller subgroup [OR 1.7 (0.9–3.5), p = 0.118].

## Discussion

In this study, we demonstrated that the presence of WMH on CT-brain imaging in LVO stroke patients is associated with worse functional outcome, but this effect does not appear to be mediated by increased risk or severity of cerebral edema or hemorrhagic transformation. It is possible that the worse functional outcome post-stroke in patients with WMH is instead a reflection of underlying brain vulnerability rather than directly leading to greater edema or HT. One study concluded that markers of brain health and atrophy, including brain parenchymal fraction, on MRI mediated poor functional outcome, whereas markers of vessel disease including enlarged perivascular spaces and WMH, were not.^21^ This supports the idea that the loss of healthy brain parenchyma is a stronger predictor of poor functional outcome, despite that atrophy may be protective against edema in that it allows the brain more room to swell. Instead, the synapse loss and diminished plasticity may lead to worse outcomes, even if the complications arising from the stroke are comparable.

Strengths of this study include using an automated segmentation method to quantify edema in a large LVO cohort from four centers with almost a thousand patients, applying three quantitative biomarkers to capture edema severity, hemispheric CSF-ratio, ΔCSF, and net water uptake. These findings suggest that the worse outcomes observed in patients with WHH are not mediated by higher risk of HT or cerebral edema as we had hypothesized and instead operates through other mechanisms.

Our findings provide further insights into the relationship between WMH and functional outcome that builds upon those previously explored. While we were able to corroborate the results of previous studies that WMH is associated with worse functional outcome,^8,10,22–24^ our results challenge the suggestion that WMH is associated with more edema after stroke,^10^ a relationship that has been sparsely studied and only using clinical edema metrics that are relatively crude and may be liable to an age bias. For example, a 2019 study evaluated CT-graded WMH and its potentially protective relationship on lateral ventricle compression,^25^ while later studies in 2022^26^ and 2024^10^ used midline shift to examine whether MRI-graded WMH correlates with risk of malignant edema. The results from these studies have each varied in findings, possibly attributed to both ventricular compression and midline shift being gross metrics of edema that indirectly measure edema at later stages and can be influenced by various factors including individual anatomical variability, pre-existing atrophy, infarct location, or thrombectomy or reperfusion status.^16,24,27,28^ For example, older patients, who are also more likely to have WMH, may experience similar degrees of edema without corresponding clinical deterioration because they have more room to swell.^29^ Consequently, by employing quantitative metrics of edema, our study provides a more nuanced powerful evaluation of the relationship between WMH and edema severity, demonstrating that the link between the two is ambiguous and is unlikely to be the basis of WMH’s association with poor functional outcome.

As for hemorrhagic transformation, some studies have found that WMH burden may be associated with higher risk for HT,^30,31^ while other populations have found weak or no association between WMH and HT, particularly in LVO or EVT populations.^32,33^ While we observed a slight protective effect of WMH for HT, this association did not appear to remain when analyzing subgroups, including those undergoing EVT, in agreement with existing literature.^32,33^ Furthermore, the risk of severe PH-type hemorrhages was not increased with WMH. Recent studies of basal ganglia strokes suggests that this heterogeneity may be due to individual markers of small vessel disease, like cerebral microbleeds, not overall WMH, as the facilitators of worse ICH, suggesting that the association of WMH with outcomes may be dependent on the stroke location and specific biological context of small vessel disease rather than the overall presence.^24^

In the quest to uncover the connection between WMH and worse functional outcome in stroke patients, we believe that our use of CT-graded WMH based on a Fazekas-derived scale serves as a particular strength to our study in terms of establishing clinical utility of these results. While WMH have historically preferentially been identified on T2-weighted MRI sequences,^1^ the standard of care in a hyperacute stroke setting is an emergent non-contrast CT brain scan,^34^ making it essential to evaluate whether CT-graded WMH can serve as a practical and prognostically meaningful tool. While this has been the inspiration for the few prior studies investigating WMH and edema on CT, ours is the first to use CT-graded WMH in conjunction with quantitative edema metrics, providing a particularly clinically relevant insight into this relationship.

Our secondary analysis of the cohort who had both imaging modalities found low interrater reliability between CT and MRI graded WMH, possibly due to the lower sensitivity of CT for detecting WMH relative to MRI.^20^ Although it may be argued that the lessened sensitivity of CT relative to MRI is a potential limitation in picking up the nuanced progression of WMH, there is evidence that CT is more specific in predicting symptomatic cerebrovascular disease.^35^ Our data, which demonstrates that CT-graded WMH has a stronger association with poor functional outcome than MRI-graded WMH, supports the suggestion that CT-graded may be more effective at picking up disease that is clinically relevant. This supports the utility for grading WMH in emergent stroke care using non-contrast head CT.

Several limitations are present in this study. First, our grading evaluation primarily relies on a combined evaluation of deep and periventricular lesions. While this scale had been previously derived and implemented,^22^ it results in less specificity in distinguishing between a patient with more severe periventricular or deep WMH. Evidence suggests that periventricular and deep white matter lesions have different pathology, with deep WMH being reflective of demyelination and gliosis and periventricular WMH being reflective of endothelial destruction.^9^ This limits our ability to draw conclusions about whether our associations with WMH, edema, functional outcome, and HT are attributable to either mechanism. Furthermore, our binary stratification of the Fazeka’s derived scale into absent-versus-any may underrepresent the full spectrum of disease severity. We had few patients with more severe grades of CT-defined WMH and so could not fully evaluate an ordinal or dose-response association of WMH severity and outcomes. Future studies could investigate the multi-level WMH scale or apply a volumetric segmentation of WMH to provide more detailed insight into how WMH severity impacts these relationships. In addition, WMH grading was performed by a single rater, with reasonable interrater reliability found for a subset. However, the choice to use the Fazekas-derived scale, which has been shown to have higher inter-rater reliability than other CT-grading methods, aimed to enhance confidence in the robustness of our approach while avoiding variability introduced by multiple graders. Finally, our cohort was limited to LVO stroke patients, a population at high risk for vascular injury, to enhance the interpretability of our findings. However, as a result, these findings may not be generalizable to all stroke populations.

## Conclusions

This study aimed to investigate how CT-graded WMH can be used to characterize the mechanistic relationship between WMH and poor functional outcome after stroke. Our hypothesis was that preexisting vascular damage associated with WMH could mediate poor functional outcome by increasing cerebral edema and hemorrhagic transformation, known indicators of poor functional outcome. However, while automated segmentation of CSF and CSF displacement on LVO stroke patients affirmed that CT-graded white matter is associated with poorer functional outcome, this effect was not mediated by worse edema formation or more hemorrhage.

## Figures and Tables

**Table 1:** Associations of Baseline Predictors and Outcome Variables with WMH (n = 782)1

Variable Name	WMH− (n = 636)	WMH+ (n = 146)	P-Value
**Patient Demographics**			
Age, years	69 (60, 78)	80 (72, 85)	< 0.001
Sex, *Male*	339 (53)	58 (40)	0.004
Hypertension	383 (60)	103 (71)	0.002
Diabetes Mellitus	107 (17)	30 (21)	0.21
Tobacco User	146 (23)	25 (17)	0.27
Admitted SBP, mm Hg	145 (131, 162)	159 (145, 174)	< 0.001
Glucose, mg/dl	112 (97, 134)	124 (104, 149)	0.009
**Imaging Characteristics and Baseline Disease Severity**			
ASPECTS	9 (7, 10)	9 (8, 10)	0.20
ASPECTS < = 7	152 (24)	24 (16)	0.22
Baseline CSF Volume, mL	171 (134, 214)	226 (183, 265)	< 0.001
Infarct Volume at 24 hours, mL	31 (8, 112)	56 (12, 134)	0.11
**Treatment and Outcomes**			
Thrombectomy	502 (79)	103 (71)	0.03
HT, any grade	186 (29)	29 (20)	0.03
HT-PH grade	67 (11)	12 (8)	0.51
CSF Ratio at 24 hours	0.78 (0.69, 0.94)	0.88 (0.72, 0.95)	0.21
ΔCSF at 24 hours	0.12 (0.02, 0.25)	0.12 (0.05, 0.25)	0.41
mRS (3–6) – 130	215 (40)	74 (63)	< 0.001
ΔNIHSS	3 (0, 8)	1 (0, 5)	0.004

Values are shown as number (percentage) or median (IQR)

1. Kruskal-Wallis Test, T-Test, Chi-Square Test

Abbreviations: ASPECTS, Alberta Stroke Program Early CT Score; BL, Baseline; CSF, Cerebrospinal Fluid; HT, Hemorrhagic Transformation; HTN, Hypertension; mRS, Modified Rankin Scale; NIHSS, National Institutes of Health Stroke Scale; PH, Parenchymal Hemorrhage; SBP, Systolic Blood Pressure; WMH, White Matter Hyperintensity.

Number of patients with missing data: hypertension 125, diabetes 127, tobacco use 187, SBP 139, glucose 134, ASPECTS 181, baseline CSF 162, infarct volume 485, thrombectomy 2, any HT 6, CSF ratio 48, mRS 130, ΔNIHSS baseline 30 / follow-up 228. Much of the missing subject data comes from one site (n = 98) that did not collect these variables.
